# No indication of *Coxiella burnetii* infection in Norwegian farmed ruminants

**DOI:** 10.1186/1746-6148-8-59

**Published:** 2012-05-20

**Authors:** Annette H Kampen, Petter Hopp, Gry M Grøneng, Ingrid Melkild, Anne Margrete Urdahl, Ann-Charlotte Karlsson, Jorun Tharaldsen

**Affiliations:** 1 Norwegian Veterinary Institute, P.O. Box 750 Sentrum, 0106, Oslo, Norway; 2 Norwegian Livestock Industry's Biosecurity Unit, P.O. Box 396 Økern, 0513, Oslo, Norway; 3 Current address: The Norwegian Farmers' Union, P.O. Box 9354 Grønland, 0135, Oslo, Norway

**Keywords:** *Coxiella burnetii*, ELISA, Freedom from disease, Q-fever, Serology, Ruminants

## Abstract

**Background:**

Infection with *Coxiella burnetii,* the cause of Q-fever, has never been detected in Norwegian animals. Recognising the increasing prevalence of the infection in neighbouring countries, the aim of the study was to perform a survey of Norwegian farmed ruminants for the prevalence of *C. burnetii* infection.

**Results:**

Milk and blood samples from more than 3450 Norwegian dairy cattle herds, 55 beef cattle herds, 348 dairy goat herds and 118 sheep flocks were serologically examined for antibodies against *C. burnetii*. All samples were negative for antibodies against *C. burnetii.* The estimated prevalences of infected herds were 0 (95% confidence interval: 0% - 0.12%), 0 (0% - 12%), 0 (0% - 1.2%) and 0 (0% - 10%) for dairy cattle herds, beef cattle herds, goat herds and sheep flocks, respectively.

**Conclusions:**

The study indicates that the prevalence of *C. burnetii* infection in farmed Norwegian ruminants is low, and it cannot be excluded that Norway is free of the infection. It would be beneficial if Norway was able to maintain the current situation. Therefore, preventive measures should be continued.

## Background

Q-fever is a zoonotic disease caused by the intracellular bacterium *Coxiella burnetii.* Most mammals and birds are susceptible to the bacterium. Natural reservoirs are a large variety of ticks and wild vertebrates, primarily rodents [1], but farmed ruminants are considered the main reservoir for transmission to humans [2,3]. Usually infected animals are asymptomatic carriers. If symptoms occur in mammals, they are most often related to the reproductive system [4]. In cattle, *C. burnetii* infection may cause metritis, reduced fertility and occasionally abortions [5-7]. In sheep and goats, abortions and stillbirths are more common than in cattle [6], and epidemics with abortion or non-viable progeny of more than 50% of pregnant animals in goat herds [8] and within groups of sheep flocks [9] have been reported. Infection with *C. burnetii* in humans is often asymptomatic, but may occur in an acute form with fever, pneumonia and/or hepatitis or in a severe chronic form with endocarditis that may be lethal if not treated [10].

Infected animals, including healthy carriers, may shed large quantities of the bacteria in amniotic fluids, placenta and vaginal excretes in relation to birth, and intermittently in milk and urine. *C. burnetii* develops spore-like stages highly resistant to environmental influence. Animals, as well as humans, usually acquire the infection by inhalation of material contaminated with the bacterium (reviewed by [6]). A varying proportion of animals develop antibodies against the bacterium, and the presence of antibodies provides evidence of a recent infection or past exposure [11]. Some individuals may harbour the organism without seroconversion [12,13]. Therefore, serological tests should not be interpreted at the individual level, but are suitable for investigation of the epidemiological status in a population or herd [11]. For screening purposes, ELISA tests are often preferred for practical reasons and because of their higher sensitivity than the complement fixation tests [11].

*C. burnetii* has a world-wide distribution. The infection is endemic in Southern and Central Europe, and the last years an apparent increase in the occurrence has been observed in Northern Europe. Since 2007, the Netherlands has experienced a concurrent epidemic in goats and humans with more than 3500 notified human cases until 2009, and extensive control measures have been implemented in the small ruminant population [14]. In Denmark, the infection is considered endemic in cattle, with the prevalence still increasing [15]. In Sweden, the bacterium was isolated from sheep placenta in 1991 [16], and 8.5% of 1000 bulk milk samples from cattle were positive for antibodies against *C. burnetii* in 2008 [17]. Finland reported their first finding of antibodies against *C. burnetii* in two heifers examined as part of an export control of cattle in 2008 [18].

Infection with *C. burnetii* has never been detected in animals in Norway, but the number of examinations for *C. burnetii* has been limited. In 1990–91, 80 aborting goats from 7 herds were examined [19] and from 2005 to 2009, 67 cattle, 12 sheep and 30 goats from 48, 3 and 1 herds respectively were serologically tested for antibodies against *C. burnetii* (unpublished data, Annette Kampen, Norwegian Veterinary Institute). In addition, ruminants have been tested in connection with import and export control. From 1989 to 2002, only twelve human Q-fever cases were reported, and ten of these individuals had acquired the infection abroad; the origin of infection for the two remaining cases was not reported [20]. Since 2002, Norwegian data on human Q-fever cases have unfortunately not been collected centrally.

Recognising the increasing prevalence of infection in neighbouring countries and the scarce knowledge of the situation in Norwegian animals, the aim of this study was to perform a serological survey of Norwegian farmed ruminants for the prevalence of *C. burnetii* infection.

## Methods

### Sampling

The study was designed as cross-sectional studies of the dairy and beef cattle, sheep and dairy goat populations with the herd as the unit of concern.

**Figure 1:**
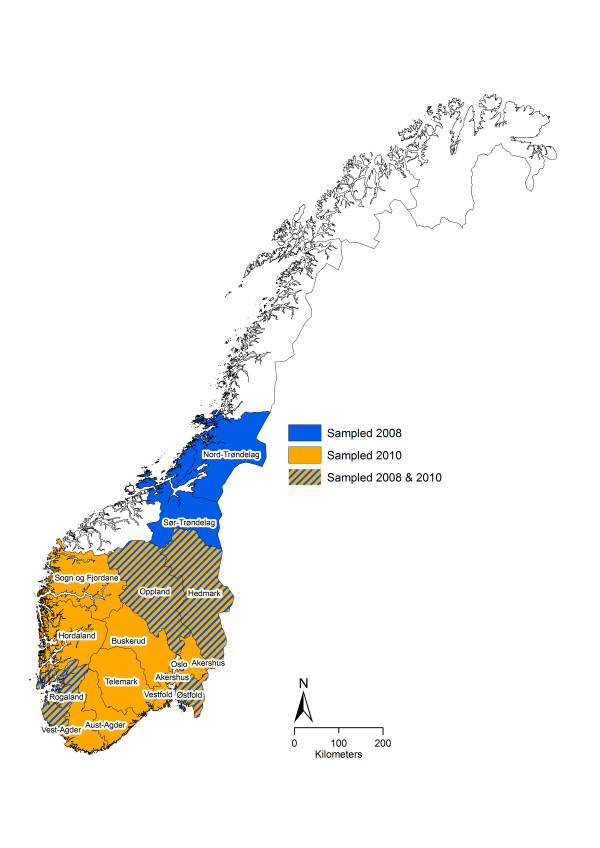
**The distribution of cattle herds sampled in a Norwegian study on seroprevalence of*Coxiella burnetii.*** The counties in which samples were collected in 2008 and 2010 are marked with blue and orange, respectively.

A list of holding IDs of dairy cattle herds delivering milk in July 2008 was obtained from the main dairy company (Tine BA) receiving milk from more than 95% of all dairy herds. In total, 6659 herds located in the counties Østfold, Hedmark, Oppland, Rogaland, Sør-Trøndelag and Nord-Trøndelag (Figure 1) were considered eligible. Bulk milk samples from 600 randomly selected dairy cattle herds were requested from the dairies. From these, samples from 460 of the selected herds were collected by the dairies and frozen before being submitted to the laboratory.

In autumn 2010, bulk milk samples from dairy cattle herds located in Southern Norway were collected as part of the surveillance and control programme for bluetongue [21]. Samples from 3317 herds originating from 13 counties (Figure 1) were collected by the dairies and frozen before being submitted to the Norwegian Veterinary Institute. Of these, sufficient material was available from 3289 different herds, and these were included in the present study.

A total of 1864 beef cattle herds from the counties Østfold, Hedmark, Oppland, Rogaland, Sør-Trøndelag and Nord-Trøndelag were registered in the register of production subsidies in July 2007 (Norwegian Agricultural Authority). Among these, 238 cattle herds had been randomly selected for the surveillance programme for enzootic bovine leukosis [22]. Only herds which had submitted at least ten blood samples by the end of October 2008 were considered eligible, leaving 85 herds eligible for the study. From these, ten blood samples from each of 55 randomly selected herds were examined in the present study.

Bulk milk samples from a total of 348 goat herds from the whole country, constituting 80% of the total dairy goat population in Norway, were included in the present study. The bulk milk samples had originally been submitted in March 2009 for examination for antibodies against caprine arthritis-encephalitis virus as part of the disease eradication programme Healthier goats [23].

From 2006 to 2008, 1179 sheep flocks from 126 different breeding groups (ram circles) had been examined in the surveillance programme for maedi/visna [24]. A random selection of 130 sheep flocks covering all the 126 ram circles was performed. Material from 118 of these flocks was available, and five blood samples from each flock were further examined in the present study.

The study was performed in agreement with Norwegian animal welfare regulations, in which the collection of blood samples is exempt from specific approval [25].

### Laboratory methods

All serum and bulk milk samples were tested using the CHEKIT Q-Fever Antibody ELISA Test Kit (IDEXX laboratories, Westbrook, WE, USA) following the manufacturer’s instructions, where the optical densities (OD) of the sample and the positive control are corrected by subtracting the OD value of the negative control, and the ratio between the sample and the positive control (S/P ratio) is calculated. The manufacturer considers an S/P ratio of <30% as negative, 30% - 40% as inconclusive and >40% as positive. In cases of inconclusive results, the samples were retested in triplets. The bulk milk samples were tested as described for single individual milk samples. The test's ability to detect a single positive animal in a bulk milk sample was estimated by examining eight positive individual milk samples that were serially diluted in serologically negative milk. The results were used to estimate the herd sensitivity of the bulk milk test (see Additional file 1).

### Data management and statistical methods

The sample information and the results of the examinations were entered into the laboratory information and management system at the Norwegian Veterinary Institute. The information on the origin of the samples collected in 2006 to 2009 was anonymized, and location was registered at the county level.

Prevalence of positive herds in the population and the corresponding 95% confidence intervals were estimated by the data analysis module in Freecalc (Survey toolbox, © Angus Cameron, Australia, 1998). As input to Freecalc were used: the total number of herds, the number of examined herds, the number of positive herds, the test specificity and the herd level sensitivity. For further details on the estimation of input parameters and the calculations, see Additional file 1.

## Results

All samples examined were negative for antibodies against *C. burnetii.* Three samples had inconclusive results in the first test round. In the following retesting, all triplets were negative and the samples were concluded negative. The estimated prevalences with corresponding 95% confidence intervals were 0 (0% - 0.12%), 0 (0% - 12%), 0 (0% - 1.2%) and 0 (0% - 10%) for dairy cattle herds, beef cattle herds, goat herds and sheep flocks, respectively (Table 1).

**Table 1:** **Results of serological examination for antibodies against*Coxiella burnetii*in Norwegian ruminants**

Herd category	Population size (herds)	Examined herds	Prevalence (%)	95% CI
Dairy cattle 2008	6659*	460	0	0-1.0
Dairy cattle 2010	6673**	3289	0	0-0.12
Beef cattle	1864*	55	0	0-12
Dairy goats	429	348	0	0-1.2
Sheep	15101	118	0	0-10

The total number of herds in the population, number of herds examined, and estimated prevalence of positive herds with 95% confidence intervals (CI) are given.

* In the six counties included in the study.

** In the 13 counties included in the study.

## Discussion

Antibodies against *C. burnetii* were not detected in the present study, giving no indication of any current or previous infection with *C. burnetii* in farmed Norwegian ruminants. If *C. burnetii* infection should be present, despite the results of this study, the prevalence in dairy cattle is at any rate low. In Norway, the farmed ruminant species cannot be considered as epidemiologically separate populations as holdings having combined production with several species and sharing of pasture between various ruminant species are common practise. Presence of *C. burnetii* infection in beef cattle herds, dairy goat herds or sheep flocks would presumably have led to infected dairy cattle as well, indicating that if the infection is present, the prevalence is probably low also in these populations.

The facts that *C. burnetii* infection has never been detected in abortion cases from ruminants [19] and that humans with *C. burnetii* infection acquired within Norway has never been reported [20], support that the prevalence of this infection in farmed ruminants is low if present at all. Furthermore, Norway has an isolated geographical location in Northern Europe, and the number of imports of domestic animals has been limited with a total of 127 cattle, 284 goats and 189 sheep imported from 2000 to 2010 [26]. Hence, the probability of introduction of infectious diseases by live animals is considered low. Currently, New Zealand is the only country considered to be free from *C. burnetii* infection based on animal surveys and no reports of indigenous human cases [11,27]. Norway has been able to obtain and maintain freedom from several other infectious diseases in ruminants, such as bovine virus diarrhoea, enzootic bovine leukosis, infectious bovine rhinotracheitis, bovine spongiform encephalopathy, tuberculosis, and ovine, caprine and bovine brucellosis [28,29]. It cannot be excluded that Norwegian farmed ruminants are free from *C. burnetii* infection as well.

In the initial study of the cattle population only six of the Norwegian counties were included. These counties comprise the majority of the cattle population, have the highest animal density, and most of the imported ruminants have been destined for holdings within this area. It was considered unlikely that the infection would be absent from these areas, if present in Norway. Therefore, the survey was targeted on these counties to increase the probability of detecting the infection. In 2010, dairy cattle from a wider geographical area were examined leaving only four counties in which dairy cattle have not been included in a survey for *C. burnetii*. These four counties comprise less than 20% of the Norwegian cattle population and have the lowest cattle density, and it was considered unlikely that the prevalence should be higher in these areas than in the examined areas.

The calculation of confidence intervals for the prevalence estimates was based on herd sensitivities that were low for all tested populations (see Additional file 1). Despite these low sensitivity estimates, the present studies documented a 95% CI of 0–0.12% in dairy cattle and 0 - 1% in goats due to the considerable number of herds tested. Important input parameters for the estimation of the herd sensitivity were the ELISA test sensitivity and the within-herd prevalence. The test is based on a tick-derived reference strain of *C. burnetii*. The use of ELISA tests based on strains isolated from ruminants has been recommended [30]. Beare *et al*. argue that there is a risk of cross-reactivity with other pathogens with the use of whole bacteria of the tick-derived reference strain, and the tests are not able to distinguish between different strains of *C. burnetii*[31]. However, in this screening, differentiation between strains was not intended, and false positive results due to non-specific cross reactions did not seem to occur. Only a limited evaluation of the ELISA test has been provided by the manufacturer, and the best available information was applied when calculating the test sensitivity for individual samples and bulk milk samples (see Additional file 1). Furthermore, the within-herd prevalence of animals serologically positive for *C. burnetii* was set to 5%, which is below the few estimates that have been reported from serological studies using ELISA (reviewed by [32]). Therefore, the authors consider the calculated confidence intervals as conservative estimates.

## Conclusions

The study indicates that the prevalence in farmed Norwegian ruminants is low, and it cannot be excluded that Norway is free of the infection. In several European countries, the number of *C. burnetii* infections has increased, resulting in an increasing number of human cases with severe disease. There is no indication that *C. burnetii* infection in Norwegian ruminants poses a source of infection for humans. It would be beneficial if Norway was able to maintain the current situation in ruminants. Preventive measures such as compulsory import control, increased awareness, biosecurity measures at farm level, and surveillance for early detection of an introduction, are potential measures to obtain this.

## Authors’ contributions

All authors have participated in the design of the study. AHK, GMG and ACK organised the collection of samples. JT was responsible for the serological testing. PH performed the statistical analysis. AHK, PH and GMG were main responsible for drafting the manuscript, and all authors gave input during the process. All authors read and approved the final manuscript.

## Supplementary Material

Additional file 1 **Estimation of 95% confidence intervals **[33-35]. Click here for file
