# Supplementary material for: No indication of Coxiella burnetii infection in Norwegian farmed ruminants
Source: BMC Vet Res. 2012 May 20;8:59. doi: 10.1186/1746-6148-8-59 (PMC3412698; doi:10.1186/1746-6148-8-59)
Supplement: Additional file 1 — Estimation of 95% confidence intervals [33-35]. [file 1746-6148-8-59-S1.pdf]

Additional file 1: Estimation of 95% confidence intervals

Additional file to the paper:

No indication of *Coxiella burnetii* infection in Norwegian farmed ruminants

Annette H. Kampen[1]*, Petter Hopp[1], Gry M. Grøneng[1], Ingrid Melkild[2§], Anne Margrete Urdahl[1], Ann-Charlotte Karlsson[1], Jorun Tharaldsen[1]

[1] Norwegian Veterinary Institute, P.O. Box 750 Sentrum, 0106 Oslo, Norway
[2] Norwegian Livestock Industry's Biosecurity Unit, P.O. Box 396 Økern, 0513 Oslo, Norway

Estimation of 95% confidence intervals

Estimation of the upper limit of the 95% confidence interval for the prevalence of herds with animals positive for antibodies against *Coxiella burnetii*.

Principles

Prevalence of positive herds in the population and the corresponding 95% confidence intervals were estimated assuming a hypergeometric distribution. The upper limit of the confidence interval was estimated using the data analysis module in Freecalc (Survey toolbox, © Angus Cameron, Australia, 1998). As input to Freecalc were used the population size, the number of examined herds, the number of positive herds, the specificity and the sensitivity (Table A1) as estimated below. The upper limit of the confidence interval (Table A1) was set to the maximum value of the "Minimum expected prevalence" on the condition that the probability of observing 0 positive reactors was less than 0.05.

Table A1. Input to Freecalc for estimation of the upper limit of the confidence interval of the prevalence estimate and the resulting upper limit of confidence interval.

Herd category	Population size (herds)	Number of examined herds	Number of positive herds	Test specificity	Herd sensitivity	Upper 95% CI
Dairy cattle 2008	6659*	460	0	1	0.66	1.0
Dairy cattle 2010	6673**	3289	0	1	0.66	0.12
Beef cattle	1864*	55	0	1	0.45	12
Dairy goats	429	348	0	1	0.57	1.2
Sheep	15101	118	0	1	0.25	10

* in the six counties included in the study
** in the 13 counties included in the study

Estimation of input values

Population size and number of examined herds

The population size was the number of herds with commercial production as registered in the Register of Production subsidies (Norwegian Agricultural Authority, Oslo). For the cattle population, only herds located in the counties included in the studies were considered when calculating the population size.

The number of examined herds was the number of herds tested in the current studies.

The number of positive herds was the number of herds with positive serological reaction, which was zero for all populations.

Test specificity
The test specificity was not known; hence, a conservative approach was used and the specificity was set to 1.

Test sensitivity for individual samples
According to the manufacturer there were 37 test positive of 37 positive animals examined [1]. The probability of obtaining 37 of 37 positive for different sensitivities is illustrated in Figure A1. The sensitivity was estimated to 0.983 which was the weighted mean for obtaining 37 positive of 37 trials.

Figure A1. The probability of acquiring 37 positive of 37 samples for various test sensitivities assuming a binomial distribution.

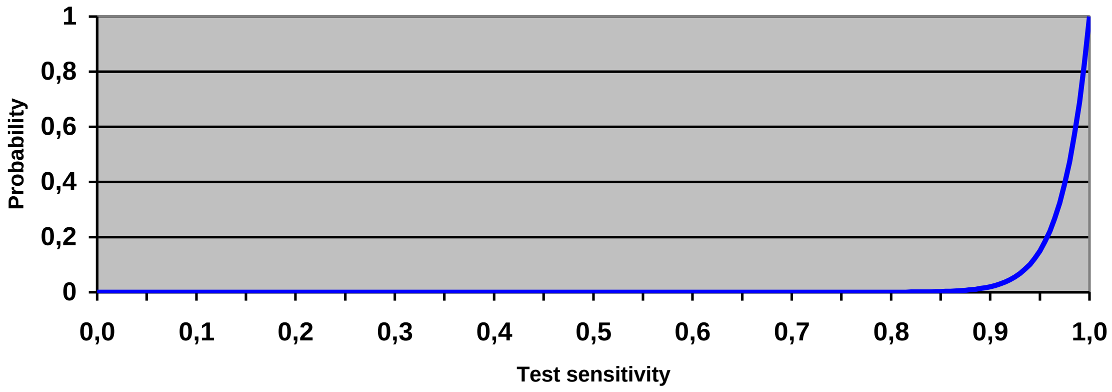

Herd sensitivity for dairy cattle and dairy goats
For dairy cattle and dairy goat herds, the herd level sensitivities (Se_H) of the test on bulk milk samples were estimated by

$$Se_H = Se_{Ind} * P(Lactating) * P(Detected)$$

where Se_{Ind} = Test sensitivity of individual samples as estimated above.
P(Lactating) = the probability that the animal is lactating at the time of sampling. For cows the average lactating period is 10 months so that on average 83% of the cows are delivering milk at a time [2]. Likewise, 92% of the goats within a herd would be lactating at the time of sampling [3].
P(Detected) = the probability that a positive sample is not diluted below the detection level.
The test's ability to detect a single positive animal in a bulk milk sample was estimated by examining eight positive individual milk samples that were serially diluted in serologically negative milk. The end-point titres were 1:10 for one sample, 1:20 for two samples, 1:40 for two samples, 1:80 for one sample, 1:320 for one sample, and 1:640 for one sample.
The population structure of dairy cattle and goat herds and the estimated number of antibody positive animals assuming that the within herd prevalence was 5% is given in Table A2. Based on this, 1 positive animal would not be diluted below detection level for herds up to 10 animals, it would be diluted below detection level in 1 of 8 in herds from 11 to 20 animals. In herds larger than 20 animals and assuming 5% antibody producing animals, the probability of detecting the animals was calculated to 0.75 (0.5 * 7 / 8 + 0.5 * 5 / 8).

Table A2. The probability that a serologically positive animal in a cattle or goat herd would be diluted below the detection level when included in the bulk milk sample.

Herd size Group	Herd size (lactating animals)	Number of antibody positive animals	P(detected)	Population proportion Cattle	Goats
1	1 - 10	1	1	0.17	0.013
2	11 - 20	1	0.875	0.49	0.017
3	21 - above	5%	0.625	0.34	0.97

With the Norwegian population structure, the mean P(Detected) for a herd was estimated by

$$\text{mean P(Detected)} = \sum \text{P(Detected}_i\text{)*(PopProp}_i\text{)}$$

where i= Herd size group. The mean P(Detected) was estimated to 0.81 and 0.63 for dairy cattle and goat herds, respectively.

The Se_H was estimated to 0.66 and 0.57 for dairy cattle and goat herds, respectively.

Herd sensitivity for beef cattle herds and sheep flocks

For beef cattle herds and sheep flocks, the herd level test sensitivity was set to 1 – the probability of detecting 0 positive reactors, assuming a hypergeometric distribution. The estimations were performed using the data analysis module in Freecalc (Survey toolbox, © Angus Cameron, Australia, 1998). As input to Freecalc were used the population size, number of examined animals, number of antibody positive animals, specificity, sensitivity (Table A3) and within-herd prevalence of 5%.

Table A3. Input to Freecalc for estimation of the herd level sensitivity for beef cattle herds and sheep flocks.

Herd category	Population size (animals)	Number of examined animals	Number of antibody positive animals	Test specificity	Individual test sensitivity
Beef cattle	22	10	0	1	0.983
Sheep	110	5	0	1	0.983

The mean herd size among the eligible herds was used as the population size of the cattle herds, i.e. 22 adult cattle. Likewise, the mean flock size among the ram circle members was used as the population size of the sheep flocks, i.e. 110 adult sheep.

From each cattle herd 10 individual blood samples were tested, and 5 individual blood samples tested from each sheep.

The herd level sensitivity for beef cattle herds was estimated to 0.45 and the flock level sensitivity for sheep flocks was estimated to 0.25.

References

1. IDEXX: **Controlling reproduction losses in herds-impact and diagnostics of four major abortive diseases.** *IDEXX Animal Health Update* 2007, **2007 (August):**1.
2. Forshell KP, Whist AC, Sogstad ÅM, Refsdal AO: *Årsmelding Helsetjenesten for storfe 2008*. Ås, Norway: Tine rådgivning; 2009. [http://storfehelse.no/2545.cms, accessed 28.02.2011.]
3. *Statistikksamling 2008*. Ås, Norway: Tine rådgivning; 2009. [http://medlem.tine.no/tp/page?id=647, accessed 28.02.2011.]
